# CD28 Individual Signaling Up-regulates Human IL-17A Expression by Promoting the Recruitment of RelA/NF-κB and STAT3 Transcription Factors on the Proximal Promoter

**DOI:** 10.3389/fimmu.2019.00864

**Published:** 2019-04-24

**Authors:** Martina Kunkl, Marta Mastrogiovanni, Nicla Porciello, Silvana Caristi, Emanuele Monteleone, Stefano Arcieri, Loretta Tuosto

**Affiliations:** ^1^Department of Biology and Biotechnology Charles Darwin, Sapienza University, Rome, Italy; ^2^Lymphocyte Cell Biology Unit, INSERM U1221, Department of Immunology, Pasteur Institute, Paris, France; ^3^Sir William Dunn School of Pathology, University of Oxford, Oxford, United Kingdom; ^4^Department of Molecular Biotechnology and Health Sciences, University of Torino, Turin, Italy; ^5^Department of Surgical Sciences, Sapienza University of Rome, Rome, Italy

**Keywords:** T lymphocytes, IL-17 A, CD28, NF-kappa B, STAT3

## Abstract

CD28 is an important co-stimulatory receptor for T lymphocytes that, in humans, delivers TCR-independent signal leading to the up-regulation of pro-inflammatory cytokines. We have recently reported that CD28 autonomous signaling induces the expression of IL-17A in peripheral CD4^+^ T lymphocytes from healthy donors, multiple sclerosis, and type 1 diabetes patients. Due to the relevance of IL-17A in the pathophysiology of several inflammatory and autoimmune diseases, we characterized the mechanisms and signaling mediators responsible for CD28-induced IL-17A expression. Here we show that CD28-mediated up-regulation of IL-17A gene expression depends on RelA/NF-κB and IL-6-associated STAT3 transcriptions factors. In particular, we found that CD28-activated RelA/NF-κB induces the expression of IL-6 that, in a positive feedback loop, mediates the activation and nuclear translocation of tyrosine phosphorylated STAT3 (pSTAT3). pSTAT3 in turn cooperates with RelA/NF-κB by binding specific sequences within the proximal promoter of human IL-17A gene, thus inducing its expression. Finally, by using specific inhibitory drugs, we also identified class 1A phosphatidylinositol 3-kinase (PI3K) as a critical upstream regulator of CD28-mediated RelA/NF-κB and STAT3 recruitments and trans-activation of IL-17A promoter. Our findings reveal a novel mechanism by which human CD28 may amplify IL-17A expression in human T lymphocytes and provide biological bases for immunotherapeutic approaches targeting CD28-associated class 1A PI3K to dampen IL-17A-mediated inflammatory response in autoimmune/inflammatory disorders.

## Introduction

IL-17-producing cells constitute a pro-inflammatory Th17 cell subset that plays critical roles in adapting immune response against extracellular pathogens and, more importantly, in the pathophysiology of several inflammatory and autoimmune diseases (1–3). The hallmark of Th17 cells is the production of the pro-inflammatory cytokines, IL-17A-F (4, 5). In particular IL-17A affects the functions of a wide range of cells. For instance, IL-17A enhances the secretion of pro-inflammatory cytokines and chemokines, favors neutrophil infiltration and increases the production of defensin and mucin by epithelial cells (6–9). Several key transcription factors, the most reliable RORC (10), cytokines, such as IL-6, TGFβ, IL-21, IL-1β, IL-23, and associated signaling mediators have been described to promote IL-17 expression and Th17 cell differentiation in both human and mouse (11–15). However, while the role of cytokines and associated signaling mediators regulating Th17 differentiation and IL-17 expression has been better defined in mouse (16–20), in humans is still controversial (14, 21, 22). Moreover, key co-stimulatory molecules regulating IL-17 expression are continuingly to be identified and may be useful for the development of new antigen non-specific immunosuppressive therapies for immune based diseases.

CD28 is an important co-stimulatory receptor that cooperates with TCR for optimal T cell activation and differentiation. CD28 contribution to IL-17 expression and Th17 differentiation has been extensively analyzed in the contest of TCR stimulation, with discrepant results depending on the strength of TCR activation, the conditioning cytokines and between human and mouse (23–31). For instance, in the mouse system, CD28 co-stimulation or associated signaling molecules have been described to either sustain (25, 26, 31) or repress TCR-mediated Th17 differentiation and functions (29, 30). Similar contrasting results have been found in humans, where CD28 co-stimulatory signals in combination with TCR activation induce (23, 28) or suppress Th17 differentiation depending on IL-1β and IL23 conditioning cytokines (27). Moreover, upon stimulation with agonistic antibodies (Ab) or with its B7.1/CD80 or B7.2/CD86 ligands expressed on the surface of professional antigen presenting cells (APC), human CD28, but not mouse CD28, is also able to act as a unique signaling receptor and to arise TCR-independent pro-inflammatory signals (32–34). Indeed, human CD28 stimulation in the absence of TCR engagement activates a NF-κB pathway in peripheral CD4^+^ T cells, thus leading to the expression and production of pro-inflammatory cytokine/chemokines (32, 35) and triggers Th17 cells to produce IL-17A (36). This CD28 pro-inflammatory activity is particularly relevant in the context of inflammatory diseases, such as multiple sclerosis (MS) and type 1 diabetes (T1D), where CD28 individual stimulation may amplify the inflammatory response by up-regulating cytokines related to the Th17 cell profile phenotype, such as IL-6, IL-21, and IL-17A (37, 38). These data suggest a role of CD28 in regulating the amplification of Th17 cells in inflammatory/autoimmune diseases. For instance, several mouse models of human inflammatory/autoimmune diseases, such as autoimmune diabetes in non-obese diabetic (NOD) mice, MS in experimental autoimmune encephalomyelitis (EAE) mice or systemic autoimmune disorders have evidenced the relevance of CD28 co-stimulatory signals (39, 40).

In the present study, we characterized the molecular mechanisms and signaling mediators regulating IL-17A expression in response to CD28 individual ligation. We found that in human CD4^+^ T cells, CD28 induced the expression of IL-6 in a NF-κB-dependent manner. IL-6 in turn acted in a positive feedback loop by inducing the activation and the nuclear translocation of tyrosine phosphorylated STAT3 (pSTAT3). pSTAT3 in synergy with RelA/NF-κB bound specific sequences within the proximal promoter of human IL-17A gene and induced its expression. Finally, by using specific inhibitory drugs, we also identified class 1A phosphatidylinositol 3-kinase (PI3K) as the upstream regulator of CD28 signals regulating IL-17A expression.

## Materials and Methods

### Cells Abs and Reagents

Human primary CD4^+^ T cells were enriched from PBMC by negative selection using an EasySep™ isolation kit (#17952, STEMCELL Technology) and cultured in RPMI-1640 supplemented with 5% human serum (Euroclone, UK), L-glutamine, penicillin and streptomycin. The purity of the sorted population was 95–99%, as evidenced by staining with anti-CD3 plus anti-CD4 Abs. Human naïve CD45RA^+^ and effector/memory CD45RO^+^ were enriched from purified CD4^+^ T cell by positive and negative selection, respectively, using a MACS anti-Phycoerythrin (PE) microbeads sorting kit (Miltenyi Biotec, Milan, Italy) after labeling with a PE-conjugated anti-CD45RA primary antibody (#130-098-184, Miltenyi Biotec, Milan, Italy). PBMCs were derived from buffy coats or anonymous healthy blood (HD) donors provided by the Policlinico Umberto I (Sapienza University of Rome, Italy). Written informed consent was obtained from blood donors and both the informed consent form and procedure was approved by the Ethics Committee of Policlinico Umberto I.

CD28-positive Jurkat T cell line has been previously described (41) and cultured in RPMI-1640 supplemented with 10% FBS (Euroclone, UK), L-glutamine, penicillin, and streptomycin. Murine L cells transfected with human B7.1/CD80 (Dap3/B7) (42), were kindly provided by Robert Lechler and Giovanna Lombardi (King's College London, UK) and cultured in DMEM supplemented with 10% FBS (Euroclone, UK), L-glutamine, penicillin, and streptomycin.

The following antibodies were used: mouse anti-human CD28 (CD28.2, #555726, 2 μg ml^−1^), mouse anti-human CD3 (UCHT1, #555330, 2 μg ml^−1^), goat anti-mouse (GAM, # 553998, 2 μg ml^−1^), anti-human CD3-PE (#555333, 1:10 dilution), anti-human CD4-APC (#130091232, 1:10 dilution), mouse anti-human STAT3 (#610189, 1:1000 dilution) (BD Biosciences); mouse anti-human Lck (3A5, # sc-433, 1:400 dilution), rabbit anti-human Lck (2102. #sc-13, 2 μg/ChIP), rabbit anti-human RNA polimerase II (N-20, #sc-899, 2 μg/ChIP), rabbit anti-human RelB (C-19, #sc-226, 1:400 dilution, 2 μg/ChIP), rabbit anti-human GAPDH (#sc-25778, 1:400 dilution) (Santa Cruz Biotechnology); mouse anti-human phosphoTyr705 STAT3 (#9138S, 1:1000 dilution), rabbit anti-human RelA (#8242S, 1:1000 dilution for western blotting, 1:100 dilution for ChIP), mouse anti-human lamin A/C (#4777S, 1:1000 dilution) (Cell Signaling Technology); rabbit anti-human lamin B1 (#ab133741, 1:1000 dilution, Abcam); mouse anti-human IL-6 (#MAB206, 10 μg ml^−1^, R&D Systems); anti-human CD45RO-FITC (#21336453, Immunotools, Germany). The following inhibitory drugs were used: MG132 (#474791, Cayman Chemical), PS1145 (#P6624, Sigma Aldrich), S31-201 (#sc-204304, Santa Cruz Biotechnology), AS605240 (#A0233, Sigma Aldrich).

### Plasmids Cell Transfection and Luciferase Assays

The NF-κB luciferase gene under the control of six thymidine kinase NF-κB sites (43) was kindly provided by J. F. Peyron (Centre Méditerranéen de Médecine Moléculaire, Nice, France). The pGL3E-hIL-17prom(-1125)-Luc construct containing the luciferase construct under the control of the 1,125 bp region upstream of the transcriptional start site of IL-17A gene (44) was from Addgene (USA). pcDNA3 expressing HA-tagged RelA and RelB have been previously described (45). Constitutive active pcDNA-STAT3C-flag construct (46) has been kindly provided by V. Poli (University of Torino, Turin, Italy).

For luciferase assays, 10^7^ cells were electroporated (at 260 V, 960 μF) in 0.5 ml RPMI-1640 supplemented with 20% FCS with 2 μg NF-κB luciferase or 5 μg pGL3E-hIL-17prom(-1125)-Luc constructs together with 5 μg pEGFP and each indicated expression vector. Twenty four hours after transfection, cells were stimulated for 6 h with Dap3/B7 cells, or anti-CD28.2, or anti-CD3 (UCHT1) plus anti-CD28 Abs. Luciferase activity was measured according to the manufacturer's instruction (#E1500, Promega). Luciferase activity determined in triplicates was expressed as arbitrary luciferase units or fold inductions after normalization to GFP values.

### Cell Stimulation and Western Blotting

Primary CD4^+^ T cells were stimulated as indicated at 37°C. At the end of incubation, total cell extracts were obtained by lysing cells for 30 min on ice in 1% Nonidet P-40 lysis buffer (150 mM NaCl, 20 mM Tris-HCl (pH 7.5), 1 mM EGTA, 1 mM MgCl_2_, 50 mM NaF, 10 mM Na_4_P_2_O_7_) in the presence of inhibitors of proteases and phosphatases (10 μg ml^−1^ leupeptin, 10 μg ml^−1^ aprotinin, 1 mM NaVO_4_, 1 mM pefabloc-SC). Nuclear extracts were prepared using NE-PER® nuclear and cytoplasmic extraction reagent (#78833, ThermoFisher Scientific). Proteins were resolved by SDS-PAGE and blotted onto nitrocellulose membranes. Blots were incubated with the indicated primary antibodies, extensively washed and after incubation with horseradish peroxidase (HRP)-labeled goat anti-rabbit (#NA934V, 1:5000 dilution) or HRP-labeled goat anti-mouse (#NA931V, 1:5000 dilution) (Amersham) developed with the enhanced chemiluminescence's detection system (GE Healthcare). Protein levels were quantified by densitometric analysis using the ImageJ 1.50i program (National Institute of Health, USA).

### Chromatin Immunoprecipitation (ChIP)

ChIP assays were performed as previously described (45). Briefly, after fixing in 1% formaldehyde, T cells were lysed for 5 min in 50 mM Tris, pH 8.0, 2 mM EDTA, 0.1% NP-40, 10% glycerol supplemented with proteases inhibitors. Nuclei were suspended in 50 mM Tris, pH 8.0, 1% SDS, and 5 mM EDTA. Chromatin was sheared by sonication, centrifuged and diluted 10 times in 50 mM Tris, pH 8.0, 0.5% NP-40, 0.2 M NaCl, 0.5 mM EDTA. After pre-clearing with a 50% suspension salmon sperm-saturated Protein-A or Protein G Sepharose beads (Amersham), lysates were incubated at 4°C overnight with anti-RelA (1:100 dilution), anti-RelB (2 μg), anti phoshoTyr705 STAT3 (pSTAT3, 1:100 dilution), anti-RNA-polimerase II (Pol II, 2 μg), or anti-Lck Ab as control. Immune complexes were collected with sperm–saturated Protein-A or Protein G Sepharose beads, washed three times with high salt buffer (20 mM Tris, pH 8.0, 0.1% SDS, 1% NP-40, 2 mM EDTA, 500 mM NaCl), and 5 times with 1x Tris/EDTA (TE). Immune complexes were extracted in 1x TE containing 1% SDS, and protein-DNA cross-links were reverted by heating at 65°C overnight. DNA was extracted by phenol-chloroform and about 1/30 of the immunoprecipitated DNA was analyzed by real-time PCR. Quantitative real-time PCR with SYBR Green Supermix (Bio-Rad) was performed for the human IL-17A promoter. Specific enrichment was calculated as previously described (47) by using the cycle threshold (Ct): 2^(CtofcontrolChIP−CtofcontrolInput)/2(CtofspecificChIP−CtofspecificInput)^. The IL-17A promoter primers used for each specific ChIP were as follow: RelA and RelB, 5′-CCAAGTTGCTTGGTAGCATGCAGGG-3′ and 5′-TTGAATATCTACTCTGCTCAAGGAA−3′; pSTAT3, 5′-TTGTTTACTTATATGATGGGAACTTGA-3′ and 5′-TTTTCCTCACAGATTCCTTGGCCA−3′; Pol II, 5′CACTGCGACACGCCACGTAAG 3′ and 5′ GGGGATGGATGAGTTTGTGCC 3′.

### Cytokine Production

Secretion of human IL-6 and IL-17A was measured from the supernatants of CD4^+^ T cells cultured for 24 h or 48 h in flat bottom 48 culture wells (3 × 10^5^ cells per well) either un-stimulated or stimulated with crosslinked anti-CD28.2 Ab (2 μg ml^−1^) by using human IL-6 (#HS HS600B) and IL-17A (#D1700) ELISA kits (R&D Technology). Data were analyzed on a Bio-Plex (Bio-Rad, Hercules, CA, USA). The assays were performed in duplicate. The sensitivity of the assay was 0.11 pg ml^−1^ for IL-6 and 15 pg ml^−1^ for IL-17A.

### Real-time PCR

Total RNA was extracted using Trizol (Thermo Fisher Scientific CA, USA) from 2 × 10^6^ cells and RNeasy MicroKit (#74004, Qiagen) from 5 × 10^5^ cells according to the manufacturer's instructions and was reverse-transcribed into cDNA by using Moloney murine leukemia virus reverse transcriptase (Invitrogen). TaqMan Universal PCR Master Mix, human IL-6, IL-17A, TGFβ, RORC, and GAPDH primer/probe sets were purchased from Applied Biosystems. The relative quantification was performed using the comparative C_T_ method. The median value of human CD4^+^ T cell stimulated with control isotype matched Ab was used as C_T_ calibrator in all comparative analyses.

### Cytotoxicity Assay

The cytotoxicity of inhibitory drugs on CD4^+^ T cells was evaluated by propidium iodide (PI) staining (10 μg ml^−1^). CD4^+^ T cells were plated at 2 × 10^6^ cells/ml in 48-well plates and treated with the indicated doses of inhibitory drugs or DMSO, as vehicle control, for 24 h. Cytotoxicity was analyzed by a BD Biosciences FACScalibur (Mountain View, CA) by quantifying the percentage of PI positive cells. Results were calculated from at least three independent experiments.

### Statistical Analysis

The sample size was chosen based on previous studies to ensure adequate power. Parametrical statistical analysis (mean and SD) was performed to evaluate differences between continuous variables through Prism 5.0 (GraphPad Software, San Diego, CA) using unpaired Student *t*-test. For multiple group comparisons, significant differences were calculated using the non-parametric Mann-Whitney *U*-test, and linear regression analyses were performed using the Pearson chi-squared test. For all tests, *p* < 0.05 were considered significant.

## Results

### CD28 Stimulation in the Absence of TCR Engagement Up-regulates IL-17A Expression in a IL-6-dependent Manner

**Figure 1:**
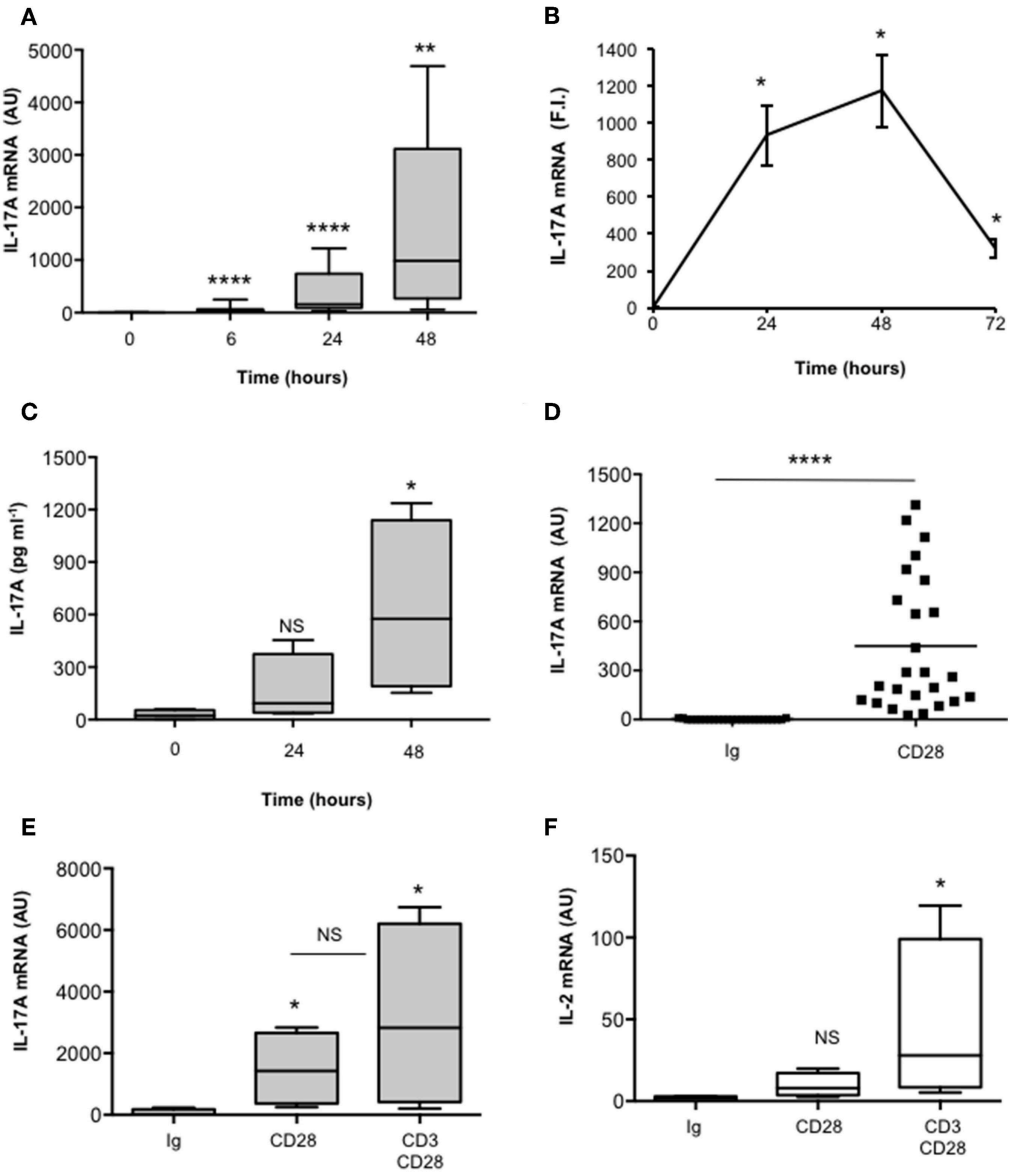
CD28 stimulation up-regulates IL-17A gene expression and production. **(A)** CD4^+^ T cells from HD subjects (*n* = 11) were stimulated for the indicated times with 2 μg ml^−1^ isotype control or anti-CD28.2 Abs. IL-17A mRNA levels were measured by real-time PCR and values, normalized to GAPDH, expressed as arbitrary units (AU). Lines represent median values and statistical significance was calculated by Mann-Whitney test. Median values: 0 h = 1, 6 h = 31, 24 h = 154, 48 h = 985. **(B)** IL-17A mRNA levels of CD4^+^ T cells stimulated for the indicated times with isotype control or anti-CD28.2 Abs. IL-17A mRNA levels were measured by real-time PCR and values, normalized to GAPDH, were expressed as fold inductions (F.I.) over the basal level of cells stimulated isotype control Ig. Data show the mean ± SD of one experiment representative of three. Statistical significance was calculated by Student *t*-test. **(C)** CD4^+^ T cells from HD subjects (*n* = 4) were stimulated for 24 or 48 h with isotype control or crosslinked anti-CD28.2 Abs. IL-17A levels in culture supernatant were measured by ELISA. Lines represent median values and statistical significance was calculated by Mann-Whitney test. Median values: 0 h = 22 pg ml^−1^, 24 h = 93 pg ml^−1^, 48 h = 575 pg ml^−1^. **(D)** IL-17A mRNA levels of CD4^+^ T cells from HD subjects (*n* = 25) stimulated for 24 h with isotype control Ig or anti-CD28.2 Abs. Lines represent median values (AU) and statistical significance was calculated by Mann-Whitney test. Median values: Ig = 2.8, CD28 = 449. **(E)** IL-17A mRNA levels of CD4^+^ T cells from HD subjects (*n* = 4) stimulated for 24 h with isotype control Ig or anti-CD28.2 or anti-CD3 (UCHT1) plus anti-CD28.2 Abs. Lines represent median values (AU) and statistical significance was calculated by Mann-Whitney test. Median values: Ig = 8, CD28 = 1427, CD3/CD28 = 2823. **(F)** IL-2 mRNA levels of CD4^+^ T cells from HD subjects (*n* = 4) stimulated for 24 h with isotype control Ig or anti-CD28.2 or anti-CD3 (UCHT1) plus anti-CD28.2 Abs. Lines represent median values (AU) and statistical significance was calculated by Mann-Whitney test. Median values: Ig = 1.7, CD28 = 8, CD3/CD28 = 28. *p < 0.05, **p < 0.01, ****p < 0.0001, calculated on cells stimulated with isotype control Ig.

We have recently found that CD28 stimulation induces the expression of IL-17A in healthy donors (HD), MS and T1D patients (37, 38). In order to better characterize the molecular mechanisms of CD28-mediated IL-17A expression, we performed a detailed kinetic analysis of IL-17A gene expression and secretion by stimulating human CD4^+^ T cells from HD with an agonistic anti-CD28 Ab (CD28.2) that has been described to bind the same epitope recognized by B7 molecules (48). CD28 stimulation by agonistic anti-CD28.2 Ab of CD4^+^ T cells from HD induced IL-17A gene expression within 6 h (Figure 1A) that further increased 24–48 h (Figures 1A,B) and decreased 72 h after stimulation (Figure 1B). CD28-induced IL-17A gene expression was also associated with a strong increase of IL-17A cytokine secretion after 48 h from stimulation (Figure 1C). As we have previously observed for other pro-inflammatory cytokines (33), CD28-induced IL-17A expression was not related to the preferential stimulation of effector/memory T cells, since no significant differences in IL-17A gene expression were observed upon stimulation of naïve (CD45RA, Figures S1A,S1C) or effector/memory (CD45RO, Figures S1B,S1C) CD4^+^ T cells with anti-CD28 Abs (Figure S1D). Furthermore, the up-regulation of IL-17A expression (Figures 1D,E) was strongly dependent on the intrinsic signaling capability of human CD28, since CD3 stimulation alone was not able to up-regulate IL-17A gene expression (Figure S1E) and no significant differences in IL-17A mRNA levels were observed when CD3 and CD28 were co-engaged compared to CD28 individual stimulation (Figure 1E). On the contrary, a high up-regulation of IL-2 mRNA was detected only in CD3 plus CD28-stimulated human CD4^+^ T cells (Figure 1F).

**Figure 2:**
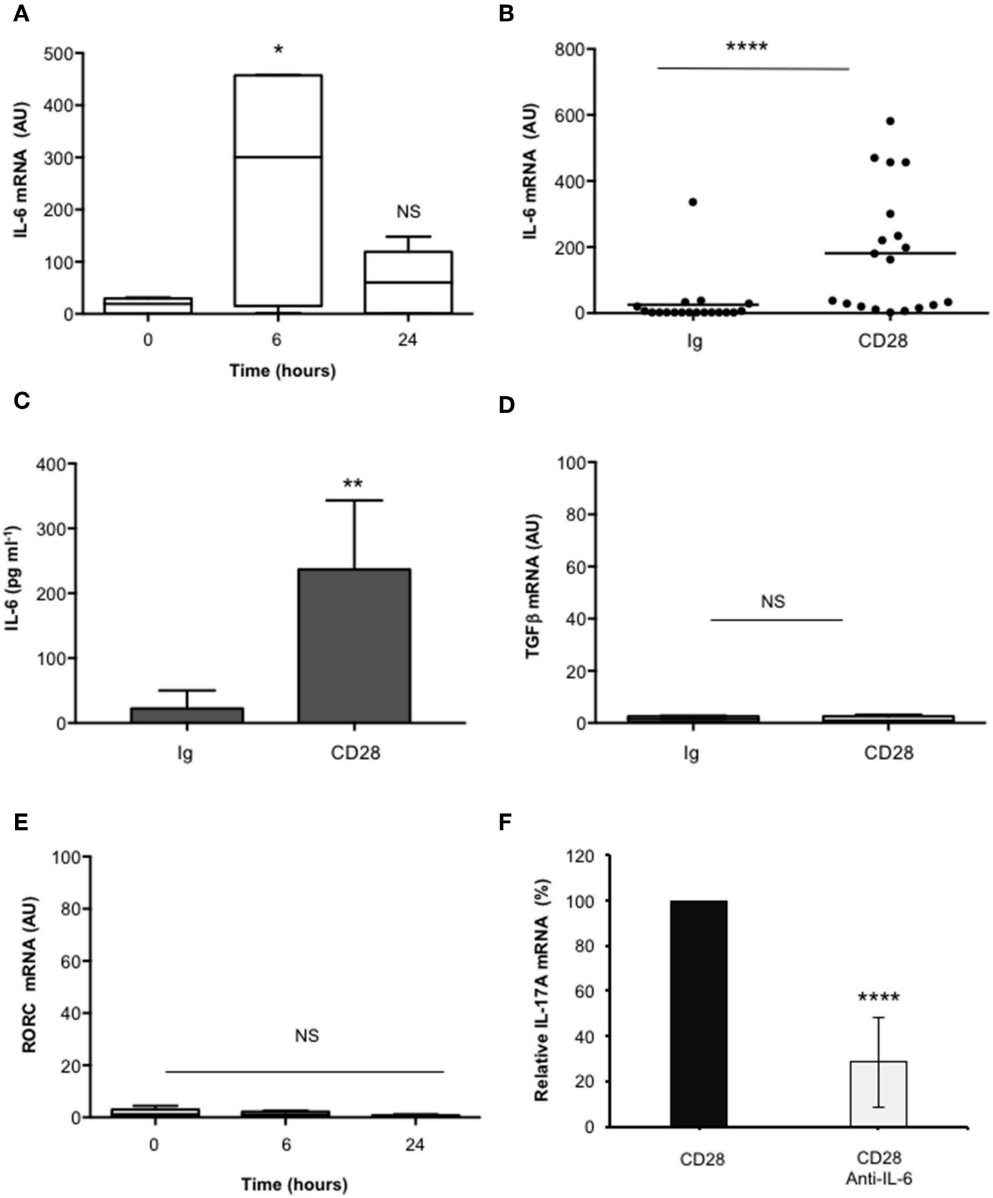
CD28-induced IL-17A gene expression depends on IL-6. **(A)** Kinetic analysis of IL-6 gene expression in CD4^+^ T cells from HD (*n* = 5) stimulated for the indicated times with isotype control Ig or anti-CD28.2 Abs. IL-6 mRNA levels were measured by real-time PCR and values, normalized to GAPDH, expressed as AU. Lines represent median values and statistical significance was calculated by Mann-Whitney test. Median values: 0 h = 19, 6 h = 300, 24 h = 60. **(B)** IL-6 mRNA levels of CD4^+^ T cells from HD (*n* = 19) stimulated for 6 h with isotype control Ig or anti-CD28.2 Abs. Lines represent median values (AU) and statistical significance was calculated by Mann-Whitney test. Median values: Ig = 1.12, CD28 = 163.1. **(C)** CD4^+^ T cells from HD subjects (*n* = 5) were stimulated for 24 h with isotype control Ig or crosslinked anti-CD28.2 Abs. IL-6 levels in culture supernatant were measured by ELISA. Bars show the mean ± SD. Statistical significance was calculated by Student *t*-test. Mean values ± SD: Ig = 22 ± 12 pg ml^−1^, CD28 = 236 ± 47 pg ml^−1^. **(D)** TGFβ mRNA levels of CD4^+^ T cells from HD (*n* = 4) stimulated for 6 h with isotype control Ig or crosslinked anti-CD28.2 Abs. Lines represent median values (AU) and statistical significance was calculated by Mann-Whitney test. Median values: Ig = 1.5, CD28 = 0.95. **(E)** RORC mRNA levels of CD4^+^ T cells from HD (*n* = 8) stimulated for the indicated times with isotype control Ig or crosslinked anti-CD28.2 Abs. Lines represent median values (AU) and statistical significance was calculated by Mann-Whitney test. Median values: 0 h = 1.1, 6 h = 1.2, 24 h = 0.4. **(F)** IL-17A mRNA levels in CD4^+^ T cells from HD (*n* = 5) stimulated for 24 h with isotype control Ig or anti-CD28.2 Abs in the presence of 10 μg ml^−1^ isotype control or neutralizing anti-IL-6 Abs. The values were normalized to GAPDH and fold inductions over isotype control Ig-stimulated cells were calculated. IL-17A mRNA values of CD28-stimulated cells were assumed as 100%. Data express the mean ± SD. Statistical significance was calculated by Student *t*-test. *p < 0.05, **p < 0.01, ****p < 0.0001, calculated on cells stimulated with isotype control Ig.

In the human system, Th17 differentiation can be mediated by both IL-6 and TGFβ that cooperate for inducing RORγt and IL-17A expression (19, 49–53). As previously reported (37), CD28 stimulation induced a strong up-regulation of IL-6 mRNAs within 6 h that decreased 24 h after stimulation (Figures 2A,B). Consistently a strong IL-6 secretion was observed after 24 h from CD28 stimulation (Figure 2C). Conversely, no significant increase of neither TGFβ (Figure 2D) nor RORC (Figure 2E) expressions were observed in CD28-stimulated primary CD4^+^ T cells. Consistently with the role of IL-6 in regulating IL-17A expression (54) and with our previous data in RRMS patients (37), the blockade of IL-6-mediated signaling by a neutralizing anti-IL6 Ab strongly inhibited (at least 70%) CD28-induced IL-17A expression in CD4^+^ T cells from HD (Figure 2F).

These data evidence that CD28-induced IL-6 cooperates in a positive feedback loop with CD28 signaling in mediating IL-17A gene expression and secretion.

### STAT3 and RelA/NF-κB Transcription Factors Regulate IL-17A Gene Expression in CD28-stimulated T Cells

**Figure 3:**
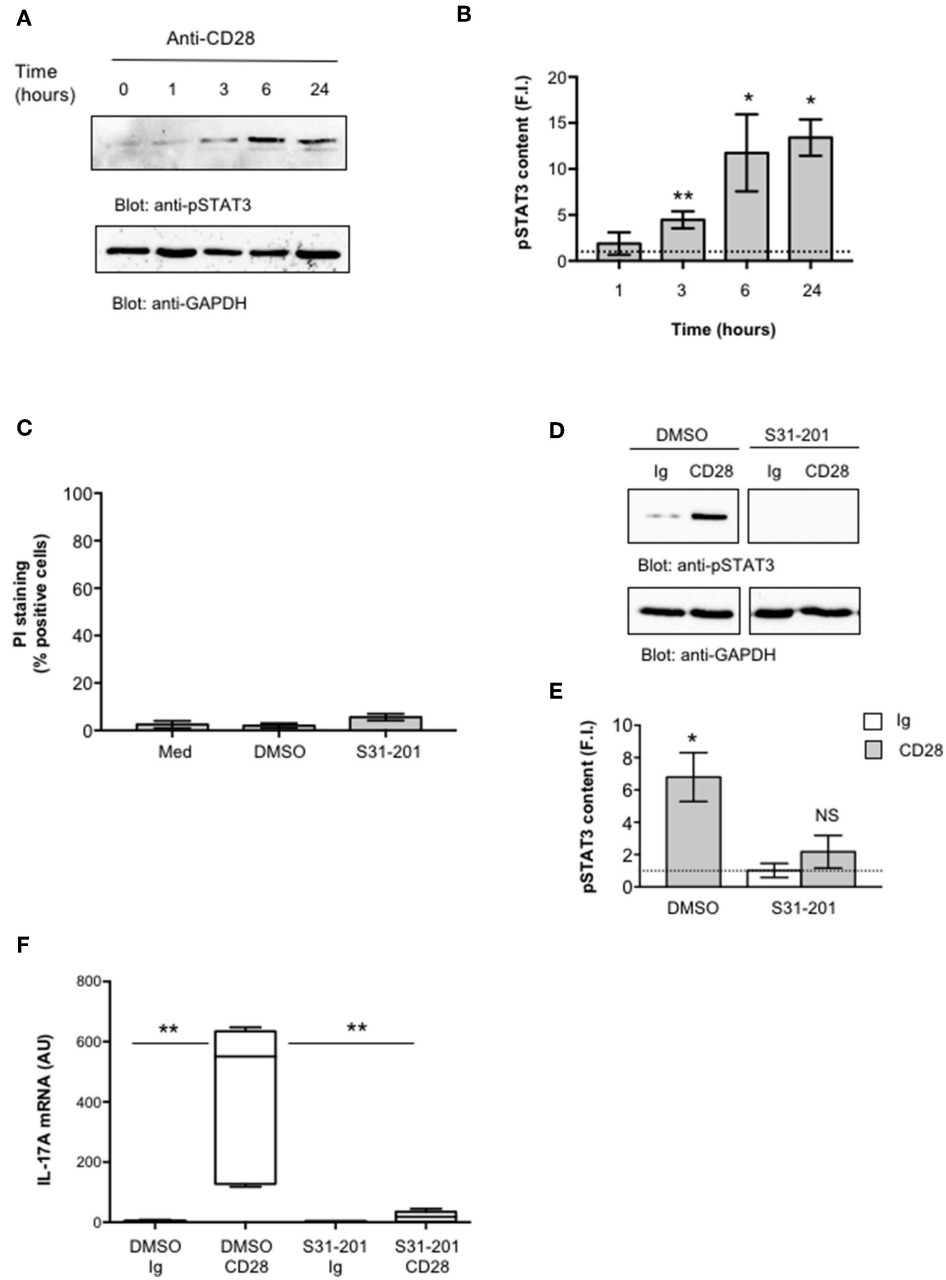
STAT3 regulates CD28-mediated up-regulation of IL-17A. **(A)** CD4^+^ T cells were stimulated for the indicated times with isotype control Ig or anti-CD28.2 Abs and STAT3 phosphorylation on tyrosine 705 (pSTAT3) and GAPDH levels were analyzed by western blotting. **(B)** pSTAT3 fold inductions (F.I.) were quantified by densitometric analysis and normalized to GAPDH levels. Bars represent mean F.I. ± SEM of three HD. *p < 0.05, **p < 0.01 calculated by Student *t*-test. **(C)** CD4^+^ T cells from HD (*n* = 4) were cultured for 24 h with medium (Med) or DMSO, as vehicle control, or 100 μM S31-201 STAT3 inhibitor. Cell death was analyzed by flow cytometry by quantifying the ability of cells to incorporate propidium iodide (PI). The percentage of PI positive cells was calculated. Results express the mean ± SEM. **(D)** Western blotting of pSTAT3 and GAPDH levels in CD4^+^ T cell treated with DMSO, as vehicle control, or S31-201 and stimulated for 6 h with isotype control Ig or anti-CD28.2 Abs. **(E)** pSTAT3 fold inductions (F.I.) were quantified by densitometric analysis and normalized to GAPDH levels. Bars represent mean F.I. ± SEM of three HD. *p < 0.05, calculated by Student *t*-test. NS, not significant. **(F)** IL-17A mRNA levels in CD4^+^ T cells from HD (*n* = 5) treated with DMSO or S31-201 and stimulated for 24 h with isotype control Ig or anti-CD28 Abs. Lines represent median values (AU) and statistical significance was calculated by Mann-Whitney test. Median values: DMSO Ig = 1, DMSO CD28 = 551, S31-201 Ig = 1.94, S31-201 CD28 = 18. **p < 0.01.

IL-6 regulates IL-17A gene expression by activating IL-6 receptor-associated STAT3 (19, 55–57). Recent studies also identified NF-κB inducing kinase (NIK) and inhibitor of NF-κB kinase α (IKKα) as important signaling factors up-regulating IL-17A gene expression (31, 58). For instance, we have previously demonstrated that CD28 stimulation in the absence of TCR engagement activates a NIK/IKKα/NF-κB2-like pathway, thus leading to the nuclear translocation of RelA-containing dimers (35, 41). Hence, we analyzed the role of STAT3 and RelA/NF-κB in regulating CD28-induced IL-17A transcription. CD4^+^ T cells were stimulated for different times with anti-CD28.2 Ab and STAT3 activation was evaluated by analyzing its level of phosphorylation on Tyr705 that has been described essential for both STAT3 dimerization and activation in response to IL-6 (59, 60). Interestingly, STAT3 phosphorylation on Tyr705 (pSTAT3) starts to augment within 3 h from CD28 stimulation and significantly increases over 6–24 h (Figure 3A, upper panel and Figure 3B). Furthermore, treatment of CD4^+^ T cells with a non-cytotoxic dose (Figure 3C) of the selective S31-201 STAT3 inhibitor (Figures 3D,E) (61, 62) strongly impaired CD28-induced IL-17A gene expression without affecting IL-17A basal mRNA levels (Figure 3F).

**Figure 4:**
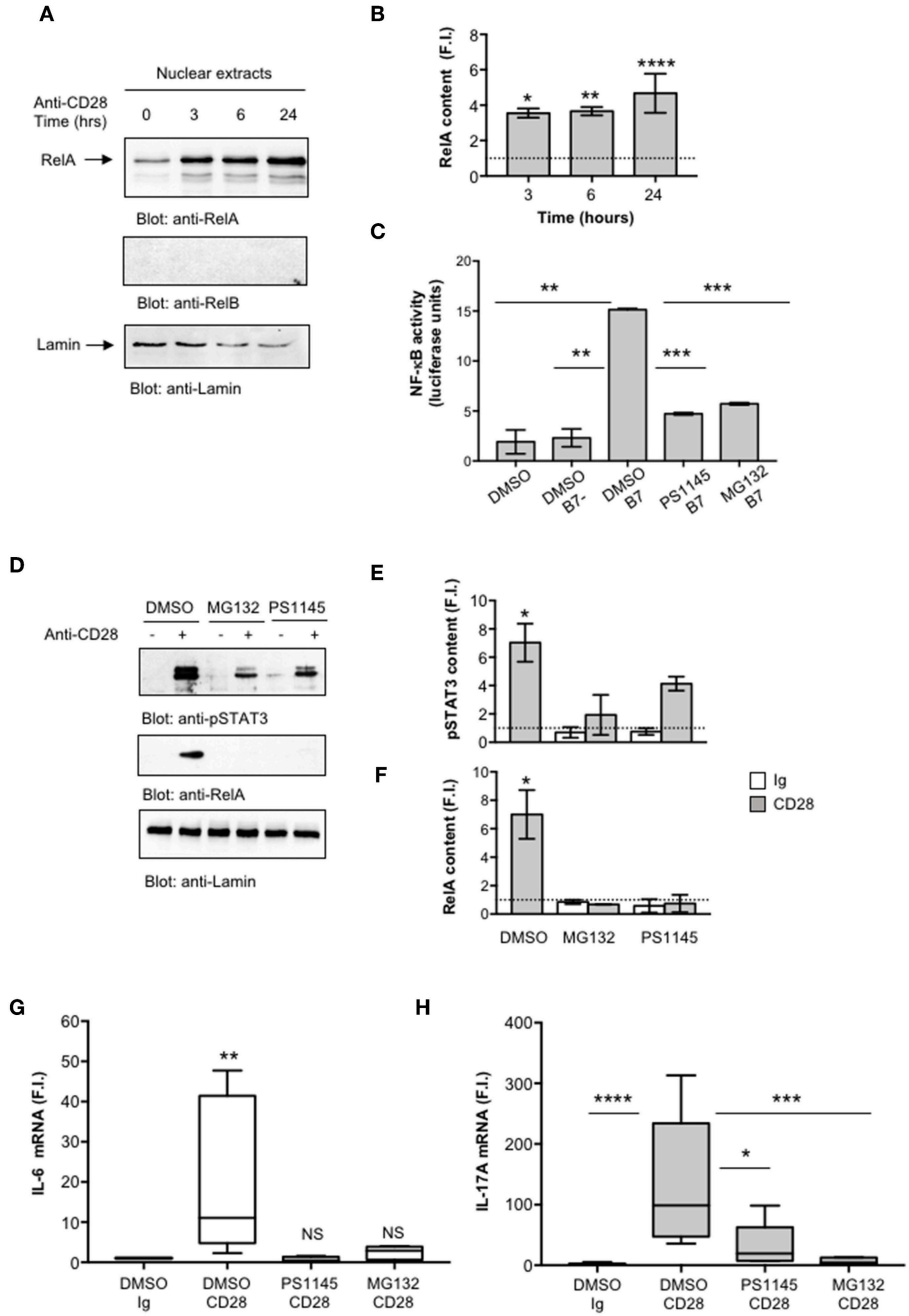
CD28-activated NF-κB regulates IL-17A expression. **(A)** CD4^+^ T cells were stimulated for the indicated times with isotype control Ig or anti-CD28.2 Abs and RelA and lamin B1 levels in nuclear extracts were analyzed by western blotting. **(B)** RelA fold inductions (F.I.) were quantified by densitometric analysis and normalized to lamin B levels. Bars represent mean F.I. ± SEM of two HD. Significance was calculated by Student *t*-test. *p < 0.05, **p < 0.01, ****p < 0.000.1 **(C)** NF-κB luciferase activity of Jurkat cells treated with DMSO, as vehicle control, or 5 μM MG132 or 10 μM PS1145 and stimulated for 6 h in the absence or presence of Dap3 (B7-) or Dap3/B7 cells (B7). The results are expressed as the mean of luciferase units ± SD after normalization to GFP values. The data are representative of three independent experiments. Significance was calculated by Student *t*-test. **p < 0.01, ***p < 0.001. Mean values ± SD: DMSO = 1.9 ± 1.1, DMSO B7 = 2.3 ± 0.9, DMSO B7 = 15.15 ± 0.12, PS1145 B7 = 4.72 ± 0.11, MG132 B7 = 5.71 ± 0.11. **(D)** Western blotting of pSTAT3, RelA, and lamin A/C levels in nuclear extracts of CD4^+^ treated with DMSO, as vehicle control, or PS1145, or MG132 and stimulated for 6 h with isotype control Ig or anti-CD28.2 Abs. pSTAT3 **(E)** and RelA **(F)** fold inductions (F.I.) were quantified by densitometric analysis and normalized to lamin B levels. Bars represent mean F.I. ± SEM of three HD. Significance was calculated by Student *t*-test. *p < 0.05. **(G,H)** IL-6 **(G)** and IL-17A **(H)** mRNA levels of CD4^+^ T cell from HD (*n* = 5) untreated or treated with DMSO, as vehicle control, or PS1145, or MG132 and stimulated for 6 h **(G)** or 24 h **(H)** with isotype control Ig or anti-CD28.2 Abs. The values were normalized to GAPDH and fold inductions (F.I.) over isotype control Ig-stimulated cells were calculated. Lines represent median values and statistical significance was calculated by Mann-Whitney test. *p < 0.05, **p < 0.01, ***p < 0.001, ****p < 0.0001. Median values: IL-6, DMSO Ig = 1, DMSO CD28 = 11, PS1145 CD28 = 0.4, MG132 CD28 = 2.8; IL-17A, DMSO Ig = 1, DMSO CD28 = 98.8, PS1145 CD28 = 19.4, MG132 CD28 = 4.3.

We next evaluated NF-κB activation by analyzing RelA translocation in nuclear extracts. As previously observed (35), CD28 stimulation induced a significant and sustained (over 24 h) increase of RelA nuclear translocation (Figures 4A,B). Consistently with our previous data (35), no significant levels of RelB were detected in nuclear extracts of CD28-stimulated CD4^+^ T cells (Figure 4A, middle panel). To assess the involvement of RelA/NF-κB in CD28-mediated IL-17A gene expression, we used the proteasome inhibitor MG-132 (63, 64) and the IKK inhibitor PS1145 (65). A CD28-positive Jurkat T cell line was transfected with NF-κB-luciferase construct and then stimulated with murine Dap3 cells (B7-) or Dap3 cells transfected with human B7.1/CD80 (B7+) in the presence or absence of non-cytotoxic doses (Figure S2A) of MG132 or PS1145. Both NF-κB inhibitors significantly impaired NF-κB transcriptional activity induced by CD28/B7 interaction (Figure 4C) as well as the nuclear translocation of pSTAT3 (Figures 4D,E) and RelA (Figures 4D,F) induced in CD4^+^ T cells by CD28 stimulation. Moreover, treatment of CD4^+^ T cells with the NF-κB inhibitors also impaired CD28-induced IL-6 (Figure 4G) and IL-17A gene expressions (Figure 4H) without affecting IL-6 (Figure S2B) and IL-17A (Figure S2C) basal expressions.

Altogether, these data strongly support a mutual cooperation of IL-6-activated pSTAT3 and RelA/NF-κB in regulating CD28-induced IL-17A gene expression.

### pSTAT3 and RelA/NF-κB Cooperate to Trans-activate the Human IL-17A Promoter in CD28-stimulated CD4^+^ T Cells

**Figure 5:**
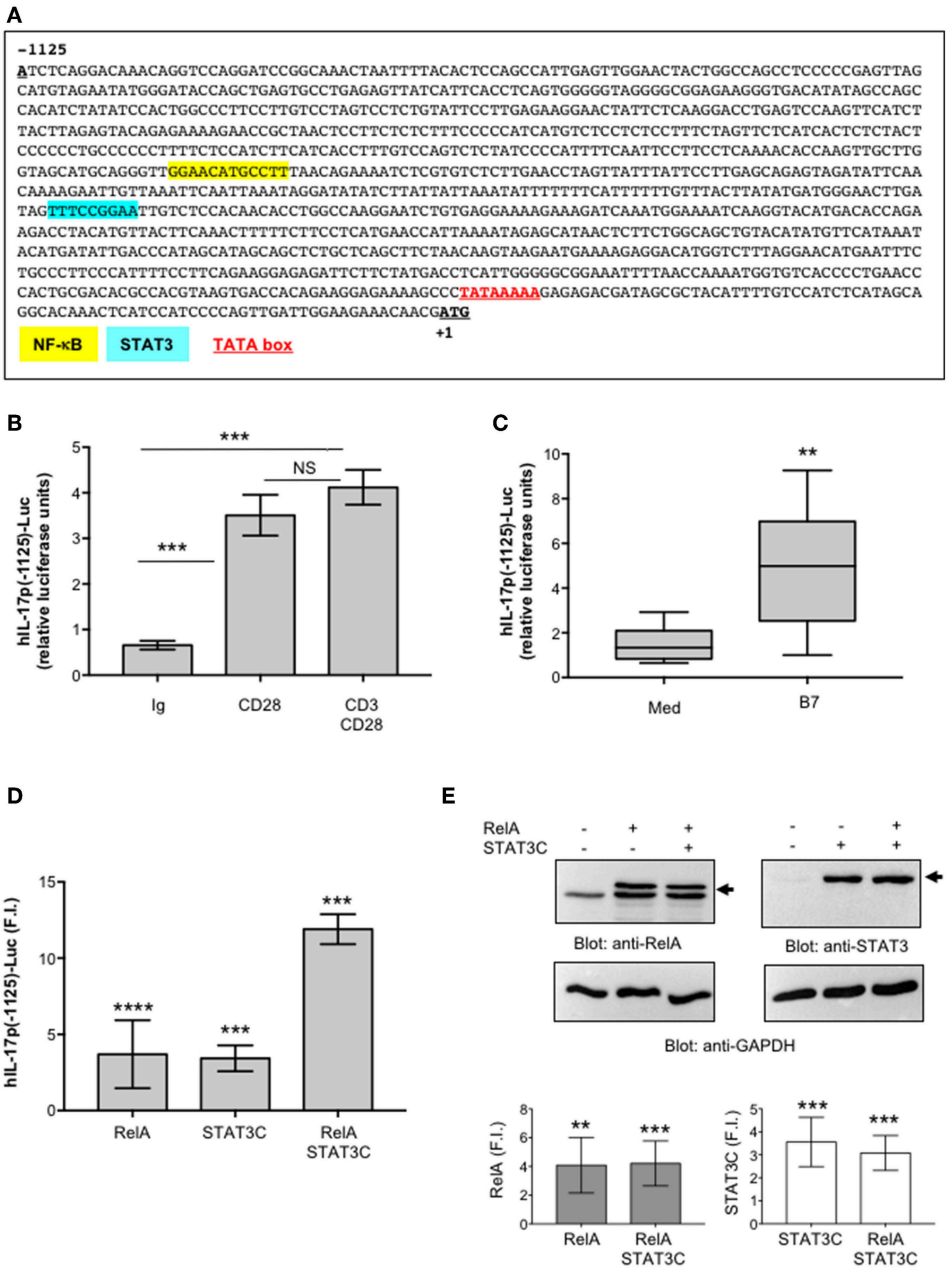
STAT3 and RelA/NF-κB transcription factors regulate CD28-mediated transactivation of IL-17A promoter. **(A)** Sequence of the 1.2 kb region upstream of the transcriptional start point of human IL-17A gene. The binding sites of NF-κB (yellow), STAT3 (light blue), and the TATA box (red) are indicated. **(B)** CD28-positive Jurkat cells were transfected with 5 μg pGL3E-hIL-17prom(-1125)-luciferase construct [hIL-17p(-1125)-Luc] and then stimulated for 6 h with isotype control Abs (Ig), or anti-CD28.2, or anti-CD3 (UCHT1) plus anti-CD28.2 Abs. The results are expressed as the mean of luciferase units ± SD. Significance was calculated by Student *t*-test. ***p < 0.001. NS, not significant. Mean ± SD: Ctr = 0.65 ± 0.09, B7 = 3.5 ± 0.53, CD28 = 3.5 ± 0.44, CD3/CD28 = 4.12 ± 0.38 The data are representative of three independent experiments. **(C)** hIL-17p(-1125)-luciferase activity of Jurkat cells stimulated for 6 h with medium (Med) or Dap3/B7 (B7) cells. Lines represent median values of eight independent experiments and statistical significance was calculated by Mann-Whitney test. **p < 0.01. Median values: Med = 1.34, B7 = 4.98. **(D)** hIL-17p(-1125)-luciferase activity of Jurkat cells transfected with EGFP construct together with vector alone (Vec) or HA-RelA, or Flag-STAT3C expression vectors alone or in combination. Fold inductions (F.I.) over the basal level of luciferase activity in cells transfected with Vec were calculated after normalization to GFP values. The results are expressed as the mean F.I. ± SD of four independent experiments. Significance was calculated by Student *t*-test. ****p < 0.0001, ***p < 0.001. Mean ± SD: RelA = 3.6 ± 2.2, STAT3C = 3.4 ± 0.9, RelA/STAT3C = 12 ± 1. **(E)** Anti-RelA, anti-STAT3 and anti-GAPDH western blotting of a Jurkat cells transfected as in **(D)**. Arrows indicate the position of HA-RelA and Flag-STAT3C. Plots represent one of four independent experiments. RelA and STAT3 fold inductions (F.I.) over the basal level of cells transfected with Vec were quantified by densitometric analysis and normalized to GAPDH levels (lower histograms). The results express the mean F.I. ± SD of four independent experiments. Significance was calculated by Student *t*-test. **p < 0.01, ***p < 0.001.

To clarify the mechanisms responsible for CD28-induced IL-17A expression, a CD28-positive Jurkat T cell line was transfected with a human IL-17A promoter-luciferase construct [hIL-17prom(-1125)-Luc] (44), containing the luciferase construct under the control of the IL-17A 5′-flanking region (-1125) upstream of the transcriptional start point (Figure 5A). Cells were then stimulated with isotype control Ig or agonistic anti-CD28.2 alone or in combination with anti-CD3 Ab. Stimulation of Jurkat cells with agonistic anti-CD28.2 Ab significantly increased (*p* < 0.001) the transcriptional activation of hIL-17p(-1125) promoter (Figure 5B). Similarly, to the data on IL-17A gene expression in primary CD4^+^ T cells (Figure 1E), no significant differences of hIL-17p(-1125) promoter trans-activation were observed when CD3 and CD28 were co-engaged compared to CD28 individual stimulation (Figure 5B). Similar results were obtained by stimulating Jurkat cells with murine L cells transfected with human B7.1/CD80 (Dap3/B7) (42) (Figure 5C). Several studies performed by our group evidenced that this experimental model perfectly mimics the physiological CD28/B7 encounter (35, 41, 45, 66).

Several STAT3 and NF-κB binding sites have been described throughout the IL-17A promoter and in the intergenic region between IL-17A and IL-17F promoters (11, 57, 67, 68). Interestingly, the proximal 1 kb region upstream of the human IL-17A gene (44) contains an optimal binding site (TTTCCGGAA) for STAT3 (69) close to the RelA/NF-κB (GGAACATGCCTT) binding site (44) (Figure 5A). Thus, a potential cooperation between STAT3 and NF-κB transcription factors in regulating CD28-induced IL-17A transcription could occur. To confirm this hypothesis, we assessed the activity of STAT3 and RelA, alone or in combination, on hIL-17p(-1125)-Luc. The single over-expression of both RelA and STAT3C constitutive active form (70) (Figure 5E) induced a significant (*p* < 0.0001) trans-activation of hIL-17A promoter at similar levels (3.5-fold inductions) (Figure 5D). The co-expression of RelA and STAT3C strongly increased hIL-17A promoter transactivation (12-fold inductions), thus indicating a cooperation of STAT3 and RelA in regulating IL-17A expression (Figure 5D).

**Figure 6:**
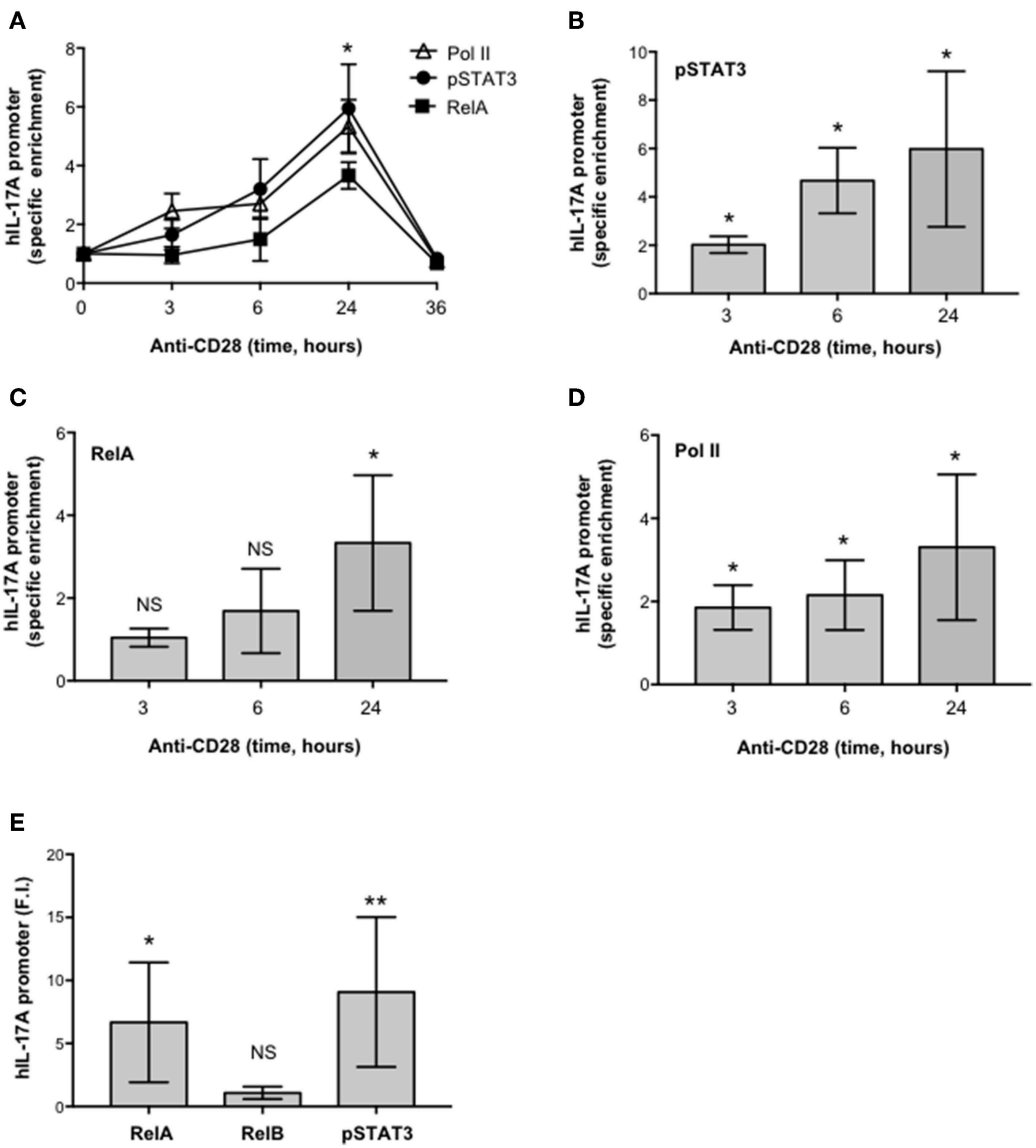
CD28 stimulation of human CD4^+^ T cells induces the selective recruitment of RelA and pSTAT3 to IL-17A promoter. **(A)** CD4^+^ T cells were stimulated for the indicated times with anti-CD28.2 Abs and anti-RelA, anti-pSTAT3, and anti-Poll II ChIPs were performed. Immunoprecipitated DNA was analyzed by real time PCR with IL-17A promoter specific primers. Specific enrichment over isotype control Abs was calculated by the Cτ method. Data express the mean ± SD of one representative experiment. Significance was calculated by Student *t*-test. **(A–D)** Real time PCR for IL-17A promoter from anti-pSTAT3 **(B)**, anti-RelA **(C)**, anti-Pol II **(D)** ChIPs performed on CD4^+^ T cells from HD (*n* = 4) stimulated for the indicated times with isotype control or anti-CD28.2 Abs. Specific enrichment over isotype control Abs was calculated by the Cτ method. Data express the mean ± SD of four independent experiments. Significance was calculated by Student *t*-test. *p < 0.05, **p < 0.01, NS, not significant. **(E)** Real time PCR for IL-17A promoter from anti-RelA, anti-RelB, and anti-pSTAT3 ChIPs performed on CD4^+^ T cells from HD (*n* = 6) stimulated for 24 h with isotype control or anti-CD28.2 Abs. Specific enrichment over negative control Abs (anti-Lck) was calculated by the Cτ method and data expressed as fold inductions (F.I.) over isotype control Ig-stimulated cells. Data express the mean F.I. ± SD. Statistical significance was calculated by Student *t*-test. *p < 0.05, **p < 0.01, NS, not significant. Mean ± SD: RelA = 6.7 ± 4.8, RelB = 1 ± 0.4, pSTAT3 = 9 ± 5.9.

To verify the physiological involvement of STAT3 and RelA in CD28-mediated trans-activation of hIL-17A promoter, we analyzed pSTAT3 and RelA recruitment on the IL-17A promoter in *ex vivo* CD4^+^ T cells stimulated with anti-CD28.2 Ab. To this aim we used ChIP assays. The kinetic analysis of both pSTAT3 and RelA recruitment on hIL-17A proximal promoter performed on CD4^+^ T cells from one HD evidenced an increase within 3–6 h with a maximum around 24 h and a decrease after 36 h (Figure 6A). The recruitment of both transcription factors was associated to CD28-induced transcriptional activation of IL-17A promoter, as evidenced by RNA polymerase II (pol II) promoter occupancy (Figure 6A). The results obtained from a larger sample size (*n* = 3) showed that pSTAT3 (Figure 6B) and Pol II (Figure 6D) were significantly recruited on the IL-17A promoter within 3 h from stimulation and persisted over 6–24 h, whereas a significant recruitment of RelA was observed after 24 h (Figure 6C). Consistently with the absence of RelB nuclear translocation (Figure 4A), no significant recruitment of RelB on the hIL-17A promoter was observed in CD28-stimulated T cells also after 24 h of stimulation compared to RelA and pSTAT3 (Figure 6E).

Altogether these data demonstrate that CD28-induced IL-17A gene expression is mediated by the cooperative recruitment and transcriptional activity of pSTAT3 and RelA on the IL-17A promoter.

### CD28-associated Class 1A PI3K Regulates pSTAT3 and RelA-mediated Transcriptional Activation of IL-17A

**Figure 7:**
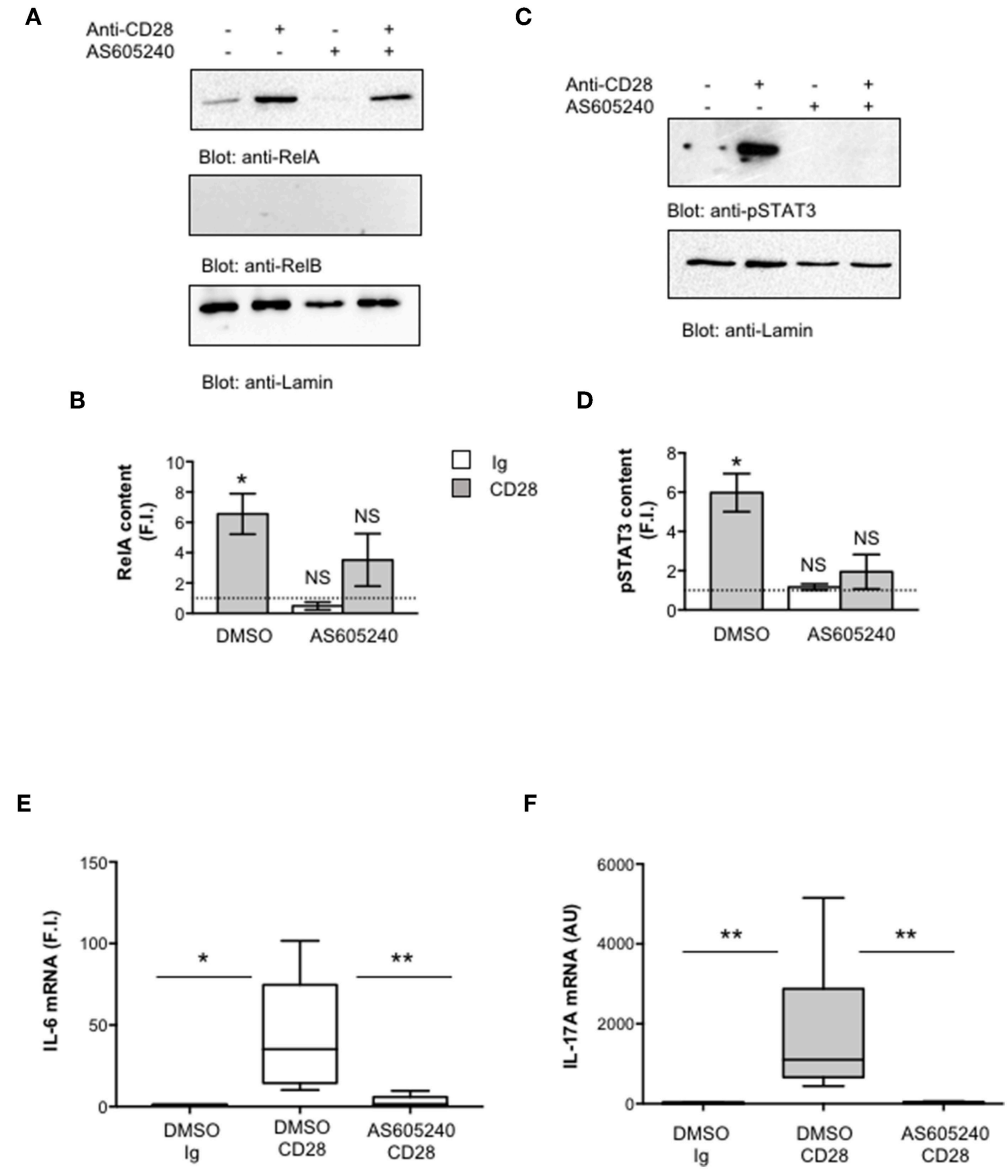
Inhibition of class 1 PI3K activity impairs pSTAT3 and RelA nuclear translocations as well as IL-6 and IL-17A gene expression induced by CD28 stimulation. **(A,B)** CD4^+^ T cells were treated with DMSO, as vehicle control, or 10 μM AS605240 and then stimulated for 3 h with isotype control Ig or anti-CD28.2 Abs. RelA, RelB, and lamin A/C **(A)** as well as pSTAT3 and lamin B1 **(B)** levels in nuclear extracts were analyzed by western blotting. RelA **(C)** and pSTAT3 **(D)** fold inductions (F.I.) were quantified by densitometric analysis and normalized to lamin levels. Bars represent mean F.I. ± SEM of three **(C)** or two **(D)** HD. Significance was calculated by Student *t*-test. *p < 0.05. NS, not significant. **(E,F)** IL-6 **(E)** and IL-17A **(F)** mRNA levels of CD4^+^ T cell from HD (*n* = 5) treated with DMSO, as vehicle control, or AS605240 and stimulated for 6 h **(E)** or 24 h **(F)** with isotype control Ig or anti-CD28.2 Abs. The values were normalized to GAPDH and expressed fold inductions (F.I.) over control Ig-stimulated cells **(E)** or AU **(F)**. Lines represent median values and statistical significance was calculated by Student *t*-test **(E)** or Mann-Whitney test **(F)**. *p < 0.05, **p < 0.01. Median values: IL-6, DMSO Ig = 1, DMSO CD28 = 35.14, AS605240 CD28 = 1.5; IL-17A, DMSO Ig = 11, DMSO CD28 = 1,776, AS605240 CD28 = 21.62.

**Figure 8:**
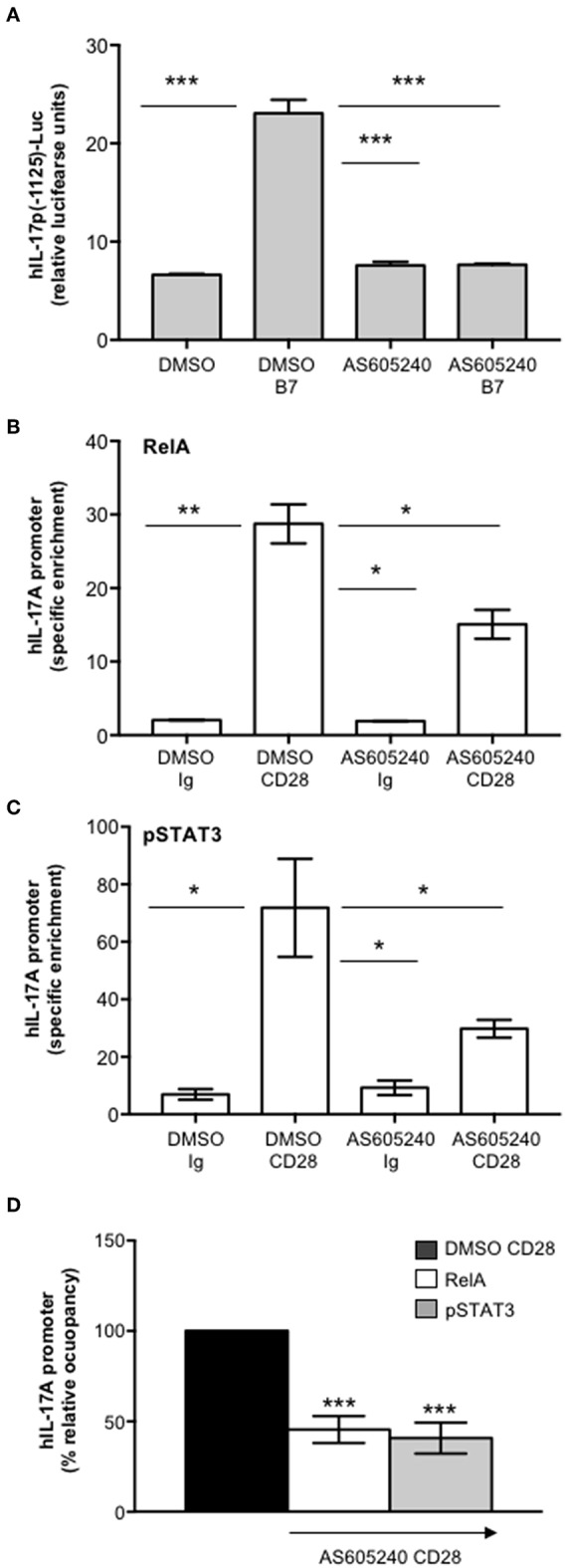
Inhibition of class 1 PI3K activity impairs CD28-induced recruitment of pSTAT3 and RelA on IL-17A promoter. **(A)** hIL-17p(-1125)-luciferase activity of Jurkat cells treated with DMSO, as vehicle control, or 10 μM AS605240 and stimulated with Dap/B7 cells (B7). Fold inductions over DMSO un-stimulated cells were calculated. The results express the mean of luciferase units ± SD on one representative experiment of three. Significance was calculated by Student *t*-test. ***p < 0.001. Mean values ± SD: DMSO = 6.6 ± 0.09, DMSO B7 = 23 ± 1.3, AS605240 = 7.6 ± 0.4, AS605240 B7 = 7.6 ± 0.08. **(B,C)** Real time PCR for IL-17A promoter from anti-RelA **(B)** and anti-pSTAT3 **(C)** ChIPs performed on CD4^+^ T cells treated with DMSO, as vehicle control, or 10 μM AS605240 and stimulated for 24 h with isotype control Ig or anti-CD28.2 Abs. Specific enrichment over negative control Abs (anti-Lck) was calculated by the Cτ method. Data express the mean ± SD of one representative experiment of three. Significance was calculated by Student *t*-test. *p < 0.05, **p < 0.01. Mean ± SD: RelA, DMSO Ig = 2 ± 0.04, DMSO CD28 = 28.74 ± 2.65, AS605240 Ig = 1.9 ± 0.06, AS605240 CD28 = 15.09 ± 1.97; pSTAT3, DMSO Ig = 6.97 ± 1.87, DMSO CD28 = 71.85 ± 17.05, AS605240 Ig = 9.25 ± 2.5, AS605240 CD28 = 29.77 ± 3; Pol II, DMSO Ig = 1.4 ± 0.26, DMSO CD28 = 7.05 ± 0.14, AS605240 Ig = 1.66 ± 0.10, AS605240 CD28 = 0.85 ± 0.18. **(D)** Real time PCR for IL-17A promoter from anti-RelA or anti-pSTAT3 ChIPs performed on CD4^+^ T cells from HD (*n* = 3) treated with DMSO, as vehicle control, or 10 μM AS605204 and stimulated for 24 h with isotype control Ig or anti-CD28.2 Abs. Specific enrichment over negative control Abs (anti-Lck) was calculated by the Cτ method and fold inductions over isotype control Ig-stimulated cells were calculated. Values of DMSO-treated and CD28-stimulated cells were assumed as 100%. Data express the mean ± SD. Statistical significance was calculated by Student *t*-test. ***p < 0.001.

One important signaling mediator of CD28 is PI3K. Indeed, CD28 binds the SH2 domain of p85 subunit of class 1A PI3K through the phosphorylated YMNM motif (71–73), thus inducing the activation of PDK1/Akt/mTORC signaling pathway that also plays a crucial role in IL-17 expression and Th17 differentiation (74–76). Treatment of CD4^+^ T cells with a non-cytotoxic dose (Figure S2A) of the class 1A PI3K inhibitor AS605240 (77), significantly impaired the nuclear translocation of both RelA (Figures 7A,B) and pSTAT3 (Figures 7C,D). Consistently with our previous data (37), the impairment of class 1A PI3K activity significantly inhibited CD28-induced transcription of IL-6 (Figure 7E) and IL-17A (Figure 7F). The trans-activation of hIL17Ap(-1125) promoter in Jurkat cells stimulated with B7-expressing cells (Figure 8A) as well as the specific recruitment of RelA (Figure 8B), pSTAT3 (Figure 8C), and Pol II (Figure 8D) on the human IL-17A promoter in CD4^+^ T cells stimulated with anti-CD28.2 Abs were also significantly impaired by AS605240 treatment.

Altogether these data support a pivotal role of CD28-associated PI3K in the transcriptional activation of IL-17A gene mediated by pSTAT3 and RelA.

## Discussion

The immunopathogenesis of several inflammatory and autoimmune diseases relies on both the amplification and persistence of pro-inflammatory IL-17-producing cells (13). Therefore, the characterization of the mechanisms and molecules regulating IL-17 expression could represent an important goal of the ongoing research in inflammation and autoimmunity.

The mechanisms regulating the differentiation of IL-17-producing cells have been largely elucidated in mice, where IL-17 can be induced by a combination of IL-6 or IL-21 and TGFβ co-signaling (16–20). In contrast to mouse T cells, in humans, several discrepancies on the factors driving and/or amplifying IL-17 expression have been reported (78). Some groups found that TGFβ alone or in combination with IL-21, IL-23, or inflammatory cytokines is required for RORγt expression and human IL-17A expression (51–53). Others evidenced that TGFβ is not essential for human Th17 differentiation and can be substituted by IL-1β (49, 79–81). More recent data by Revu et al. showed that IL-23 and IL-1β promote IL-17 production and human Th17 differentiation in the presence of TCR engagement and in the absence of CD28 stimulation, thus confirming also in the human system (27) previous data on a suppressive role of murine CD28 in Th17 cell differentiation (28, 29). Herewith, we evidence a novel role of human CD28 in inducing IL-17A expression and production in the absence of TCR engagement and conditioning cytokines (Figure 1). The transcriptional activation of IL-17A in response to CD28 was independent of either TGFβ or RORγτ, since no up-regulation of neither TGFβ (Figure 2D) nor RORC (Figure 2E) was observed in CD28-stimulated cells. By contrast, CD28-induced up-regulation of IL-17A depended on IL-6, as demonstrated by the strong inhibitory effects exerted by culturing T cells in the presence of neutralizing anti-IL-6 Ab (Figure 2F).

The ability of human CD28 to induce the expression of IL-6 and other inflammatory cytokines has been amply described (33–35, 37, 38). The kinetic analysis of CD28-induced IL-6 expression evidenced that IL-6 gene expression and secretion occurred earliest (Figures 2A–C) compared to IL-17A (Figures 1A–D) and was dependent on CD28-induced NF-κB activity (Figure 4E). These data are consistent with those from Serada et al. showing that IL-6 blockade inhibited myelin-specific Th17 cells *in vivo* (82). Since IL-6 may regulate IL-17A gene expression by activating IL-6 receptor-associated STAT3 (19, 55–57), these data strongly support a role of IL-6-induced STAT3 activation in regulating CD28-mediated IL-17A expression. For instance, a strong and persistent increase of STAT3 phosphorylation (over 24 h) was observed in CD4^+^ T cells within 3 h from CD28 stimulation (Figure 3A) and CD28-induced IL-17A expression was abrogated by the selective STAT3 inhibitor, S31-201 (Figures 3C,D). STAT3 is an important transcription factor for both murine and human Th17 cell differentiation. Deletion of STAT3 in T cells abrogates Th17 differentiation (56, 83), whereas the overexpression of constitutively active STAT3 is sufficient for inducing the development of IL-17-producing cells (84). More recent data in mouse and human systems confirmed a critical role of STAT3 in regulating the transcription of several genes involved in Th17 differentiation and functions (57, 67). Several STAT3 binding sites have been identified throughout the IL-17A promoter (11, 56, 85) and in the proximal 1-kb region upstream of the human IL-17A gene (44) (Figure 5A). For instance, we found that CD28 stimulation by either agonistic Ab or its natural ligand B7.1 strongly up-regulated the 1-kb proximal human IL-17 promoter in a STAT3-dependent manner (Figure 5). These data are in contrast with those from Liu et al. who did not find any transactivation of the proximal IL-17A promoter following CD28 stimulation alone (44). These discrepancies could be related to the distinct binding properties and stimulating activities of the anti-CD28 Ab used by Lin et al. compared to the natural B7 ligand or the agonistic anti-CD28.2 Ab (86). Moreover, our ChIP data showing the binding of STAT3 to the proximal human IL-17A promoter (Figure 6B) support a direct function of STAT3 in regulating IL-17A gene expression in CD28-stimulated CD4^+^ T cells.

The pro-inflammatory properties of human CD28 rely on its unique intrinsic capability to activate NF-κB (32–34, 87). The NF-κB family consists of five members including NF-κB1 (p50 and its precursor p105), NF-κB2 (p52 and its precursor p100), RelA (p65), RelB, and c-Rel. RelA, c-Rel and RelB contain a transactivation domain and form transcriptionally active heterodimers in association with p50 and/or p52 (88). Among the NF-κB subunits, c-Rel and RelA have been described to play a crucial role in Th17 differentiation. In mouse T cells, deficient in c-Rel or RelA, Th17 differentiation and functions were significantly compromised (30). On the contrary, RelB-deficient CD4^+^ T cells showed enhanced IL-17 expression (89). More recent findings identified NIK and IKKα as important signaling factors that regulate IL-17A gene expression and Th17 differentiation (31, 58). Interestingly, our previous data demonstrated that CD28 individual signaling leads to a non-canonical NF-κB2-like cascade by recruiting and activating NIK and IKKα (41, 90). As previously demonstrated (35), CD28 stimulation induces a persistent nuclear translocation of RelA, but not RelB (Figures 4A, 7A) nor c-Rel (35). CD28-induced RelA/NF-κB activity was crucial for both IL-6 expression (Figure 4G) and IL-6-associated STAT3 activation (Figures 4D,E) as well as for IL-17A expression (Figure 4H). The ability of CD28 autonomous stimulation to induce IL-17A production was also evidenced by Santarlasci et al., who demonstrated that CD28 stimulation alone triggers Th17 T cell clones to produce IL-17A in a NF-κB-dependent manner (36). We extended these data by evidencing the dual pivotal role of RelA/NF-κB in CD28-mediated up-regulation of IL-17A expression. Early during CD28-stimulation, RelA/NF-κB mediates the transcription of IL-6 and subsequent IL-6-dependent STAT3 activation (Figures 4D,F). Later, persistent RelA/NF-κB subunits bind the NF-κB consensus site at position−643 of the proximal human IL-17A promoter (44), thus cooperating with STAT3 for inducing IL-17A transcription (Figures 5, 6). For instance, an optimal recruitment of both STAT3 and RelA on the human IL-17A promoter was observed 24 h after CD28 stimulation (Figure 6). Interestingly, Whitley et al. have recently shown that, in mouse T cells, RelA/NF-κB regulates IL-17 expression in cooperation with STAT3 by binding specific regulatory elements. However, in contrast to human IL-17A gene (44), the mouse NF-κB and STAT3 binding sites are located in distal cis-regulatory elements of *Il17a-Il17f* loci (68), but not in the proximal promoter, thus suggesting that the regulation of IL-17A transcription may differ between human and mouse.

One important signaling mediator of CD28 is the PI3K. Indeed, CD28 binds the SH2 domain of p85 subunit of class 1A PI3K (p110δ) through the phosphorylated YMNM motif (73). Class 1A PI3K phosphorylates inositol phospholipids on carbon atom 3, thus generating the phospholipids PI 3,4-biphosphate (PIP2) and PI 3,4,5-triphosphate (PIP3) that in turn recruits Akt and PDK1 to the membrane, thus favoring their activation (71–73, 91, 92). The PI3K/PDK1/Akt pathway is essential for both CD28-mediated co-stimulatory signals and pro-inflammatory functions as well as for NF-κB activation (32, 93, 94). Consistently, we found that the selective inhibition of class 1 PI3K activity, impaired CD28 unique signaling leading to RelA nuclear translocation and NF-κB transcriptional activity as well as IL-6 expression and IL-6-dependent STAT3 phosphorylation and nuclear translocation (Figure 7). Class 1 PI3K/PDK1/Akt pathway also governs Th17 differentiation via multiple mechanisms (75), including STAT3 phosphorylation (74) and the nuclear translocation of RORγτ (95). Recent data from Way et al. showed that PI3K p110δ inhibition impairs IL-17 production form CD28-costimulated Th17 effector cells (96). Our data further evidence a novel role of class 1A PI3K in regulating CD28-induced recruitment of both RelA and pSTAT3 on the IL-17A promoter and on its transcriptional activation (Figure 8).

The identification of a new IL-6-STAT3/NF-κB axis, often altered in several immune-based diseases (97, 98), linking CD28 to IL-17A expression provide biological bases for immunotherapeutic approaches targeting CD28 and/or associated signaling mediators in order to dampen the inflammatory response in autoimmune/inflammatory disorders (39).

## Ethics Statement

This study was carried out in accordance with the recommendation of Ethical Committee of the Policlinico Umberto I (Sapienza University, Rome, Italy). All subjects gave written informed consent in accordance with the Declaration of Helsinki.

## Author Contributions

MK and MM performed the research and data analyses. NP and SC performed parts of the research. SA contributed samples for the study. EM contributed with STAT3C construct and commented on manuscript. LT designed the study, coordinated the work, and wrote the manuscript.

### Conflict of Interest Statement

The authors declare that the research was conducted in the absence of any commercial or financial relationships that could be construed as a potential conflict of interest.
