# Supplementary material for: CD28 Individual Signaling Up-regulates Human IL-17A Expression by Promoting the Recruitment of RelA/NF-κB and STAT3 Transcription Factors on the Proximal Promoter
Source: Front Immunol. 2019 Apr 24;10:864. doi: 10.3389/fimmu.2019.00864 (PMC6491678; doi:10.3389/fimmu.2019.00864)
Supplement: Supplementary file 1 [file Data_Sheet_1.PDF]

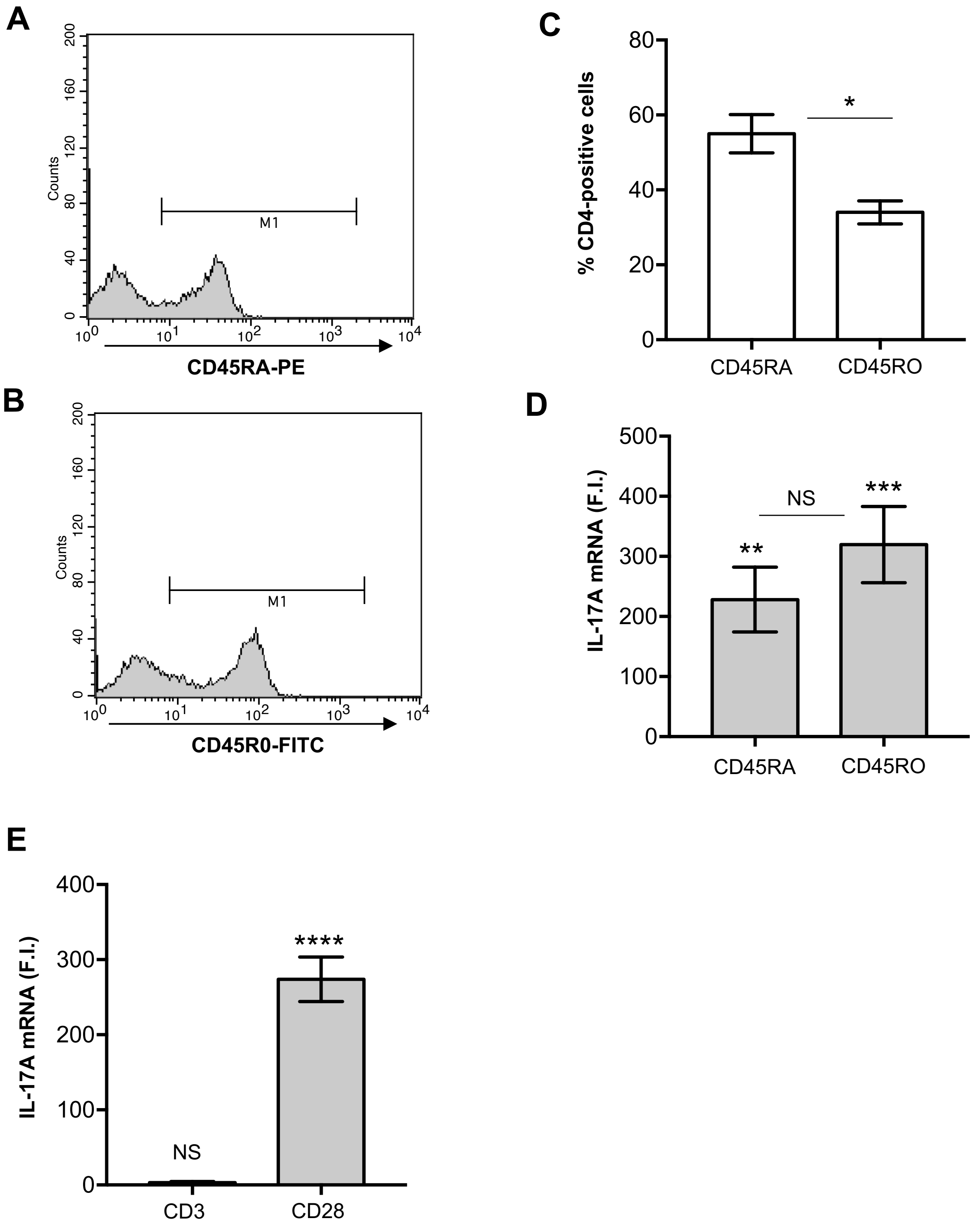

Fig. S1. (**A, B)** FACS analysis of CD45RA (**A**) and CD45RO expression of human peripheral blood $CD4^+$ T cells (**B**). (**C**) The percentage of $CD4^+$ T cells expressing CD45RA or CD45RO from HD (n = 3) was calculated. The results express mean percentage of positive cells ± SEM. (**D**) IL-17A mRNA levels in naïve (CD45RA) or effector/memory (CD45RO) $CD4^+$ T cells from HD (n = 3) stimulated for 24 h with 2 µg/ml of crosslinked CD28.2 Abs or isotype control IgG. IL-17A mRNA levels were measured by real-time PCR after normalization to GAPDH. Fold inductions (F.I.) were calculated over the basal level of cells stimulated with isotype control IgG. Bars indicate mean F.I. ± SEM. (**E**) IL-17A mRNA levels of $CD4^+$ T cells from HD (n = 3) stimulated for 24 h with 2 µg/ml of crosslinked anti-CD28.2 or anti-CD3 (UCHT1) Abs or isotype control IgG. Cytokine mRNA levels were measured by real-time PCR after normalization to GAPDH. Fold inductions (F.I.) were calculated over the basal level of cells stimulated with isotype control IgG. Bars indicate mean F.I. ± SEM. (*) $p < 0.05$, (**) $p < 0.01$, (***) $p < 0.001$, (***) $p < 0.001$ calculated by Student's t test. NS = not significant.
