# Supplementary material for: CD28 Individual Signaling Up-regulates Human IL-17A Expression by Promoting the Recruitment of RelA/NF-κB and STAT3 Transcription Factors on the Proximal Promoter
Source: Front Immunol. 2019 Apr 24;10:864. doi: 10.3389/fimmu.2019.00864 (PMC6491678; doi:10.3389/fimmu.2019.00864)
Supplement: Supplementary file 2 [file Data_Sheet_2.PDF]

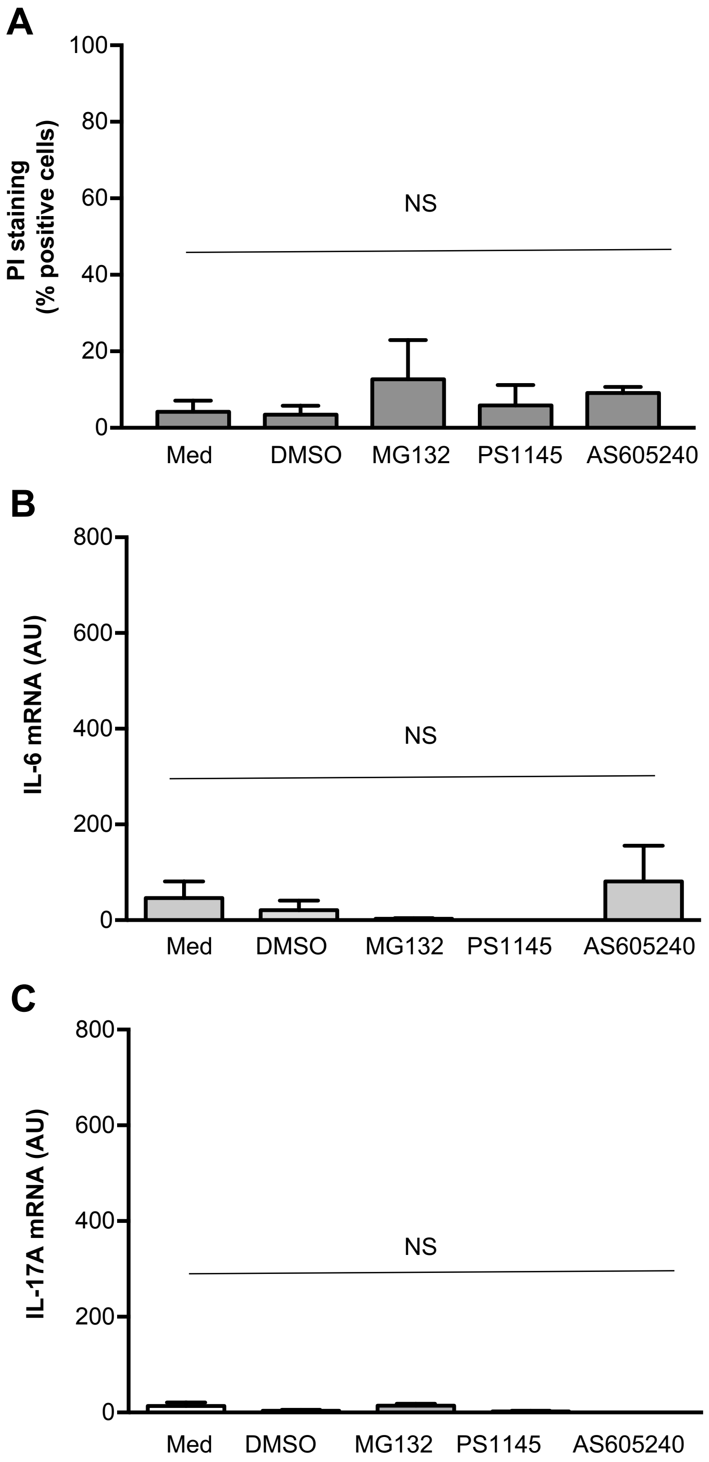

Fig. S2. NF-κB and class 1 PI3K inhibitory drugs did not affect neither cell viability nor IL-17A and IL-6 basal expression. (**A**) CD4^{+} T cells from HD (n = 3) were cultured for 24 h with medium (Med) or DMSO, as vehicle control, or 5 μM MG132, or 10 μM PS1145, or 10 μM AS605240. Cell death was analysed by flow cytometry by quantifying the ability of cells to incorporate propidium iodide (PI). The percentage of PI positive cells was calculated. Results express the mean ± SD and statistical significance was calculated by Mann-Whitney test. (**B, C**) CD4^{+} T cells from HD (n = 3) were cultured with medium (Med) or DMSO, as vehicle control, or MG132, or PS1145, or AS605240 and after 6 h (**B**) or 24 h (**C**) IL-6 (**B**) and IL-17A (**C**) mRNA levels were measured by real time PCR. Lines represent Results express the mean (AU) ± SD and statistical significance was calculated by Mann-Whitney test. NS = not significant
